# Immunological Significance of HMGB1 Post-Translational Modification and Redox Biology

**DOI:** 10.3389/fimmu.2020.01189

**Published:** 2020-06-10

**Authors:** Man Sup Kwak, Hee Sue Kim, Bin Lee, Young Hun Kim, Myoungsun Son, Jeon-Soo Shin

**Affiliations:** ^1^Department of Microbiology, Yonsei University College of Medicine, Seoul, South Korea; ^2^Institute for Immunology and Immunological Diseases, Yonsei University College of Medicine, Seoul, South Korea; ^3^Center for Autoimmune Musculoskeletal and Hematopoietic Diseases, Institute of Molecular Medicine, The Feinstein Institutes for Medical Research, Manhasset, NY, United States; ^4^Severance Biomedical Science Institute, Yonsei University College of Medicine, Seoul, South Korea; ^5^Center for Nanomedicine, Institute for Basic Science (IBS), Yonsei University, Seoul, South Korea

**Keywords:** high mobility group box1 (HMGB1), oxidation, inflammation, therapeutic target, danger-associated molecular pattern (DAMP)

## Abstract

Most extracellular proteins are secreted via the classical endoplasmic reticulum (ER)/Golgi-dependent secretion pathway; however, some proteins, including a few danger-associated molecular patterns (DAMPs), are secreted via non-classical ER/Golgi-independent secretion pathways. The evolutionarily conserved high mobility group box1 (HMGB1) is a ubiquitous nuclear protein that can be released by almost all cell types. HMGB1 lacks signal peptide and utilizes diverse non-canonical secretion mechanisms for its extracellular export. Although the post-translational modifications of HMGB1 were demonstrated, the oxidation of HMGB1 and secretion mechanisms are not highlighted yet. We currently investigated that peroxiredoxins I and II (PrxI/II) induce the intramolecular disulfide bond formation of HMGB1 in the nucleus. Disulfide HMGB1 is preferentially transported out of the nucleus by binding to the nuclear exportin chromosome-region maintenance 1 (CRM1). We determined the kinetics of HMGB1 oxidation in bone marrow-derived macrophage as early as a few minutes after lipopolysaccharide treatment, peaking at 4 h while disulfide HMGB1 accumulation was observed within the cells, starting to secrete in the late time point. We have shown that HMGB1 oxidation status, which is known to determine the biological activity in extracellular HMGB1, is crucial for the secretion of HMGB1 from the nucleus. This review summarizes selected aspects of HMGB1 redox biology relevant to the induction and propagation of inflammatory diseases. We implicate the immunological significance and the need for novel HMGB1 inhibitors through mechanism-based studies.

## Introduction

High mobility group box 1 (HMGB1) is an abundant non-histone nuclear protein that was discovered over four decades ago. The protein was isolated from calf thymus chromatin by 0.35 M NaCl extraction (1) and was then biochemically characterized (2). Based on its mobility during polyacrylamide gel electrophoresis, Goodwin et al. termed the proteins “high mobility group,” or HMG proteins; however, the group of proteins that migrated more slowly during polyacrylamide gel electrophoresis were termed “low-mobility group” proteins. HMG proteins were therefore divided into two groups based on their molecular weight: higher—HMG-1 and HMG-2 (now HMGB1 and HMGB2), lower—HMG-14 and HMG-17 (now HMGN1 and HMGN2), and HMG-I, -Y (now HMGA1a and HMGA1b) (3–9).

HMG proteins are categorized into three superfamilies based on the specific functional domains or motifs via which they recognize individual DNA structures on chromatin: HMGA, HMGB, and HMGN. Proteins in the HMGA family contain an AT-hook, which is a DNA-binding motif with a preference for A/T rich regions. In contrast, those in the HMGB family contain A-box and B-box functional motifs and those in the HMGN family contain a nucleosomal binding domain (NBD). HMGB proteins are ubiquitous and abundant in most cells and can bind to DNA without sequence specificity (10–12).

**Figure 1:**
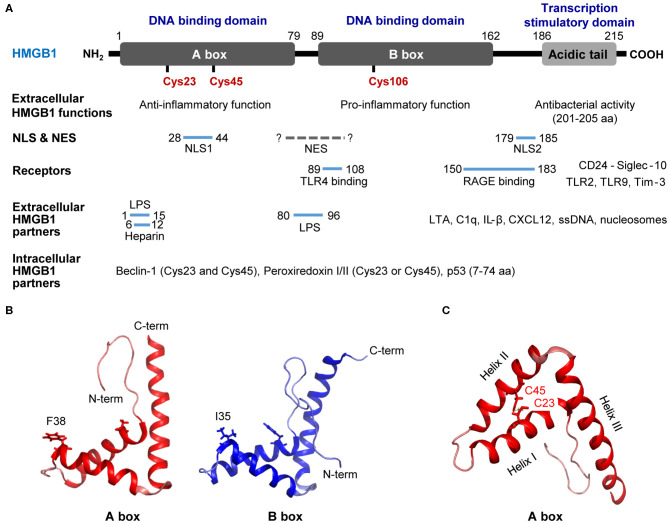
Structure of HMGB1. **(A)** Detailed summary of various domains and motifs in HMGB1. **(B)** Signature L-loop structure of the A-box and B-box structures of HMGB1. **(C)** Overall structure of HMGB1 showing the functional A-box cysteines (C23 and C45) and their geometric distribution in HMGB1. A-box and B-box structures modified based on 2YRQ [Protein Data Bank (PDB) IP: 2yrq] using PyMol. NLS, nuclear localization sequence; NES, nuclear export signal; TLR4, toll-like receptor 4; RAGE, receptor for advanced glycation endproducts; Tim-3, T-cell immunoglobulin and mucin domain-containing protein-3; LPS, lipopolysaccharide; LTA, lipoteichoic acid; C1q, complement component 1q; IL-1β, interleukin 1β; CXCL12, C-X-C motif chemokine 12; F, phenylalanine; I, isoleucine; C, cysteine; C-term, C-terminal; N-term, N-terminal. Solid line; known binding domain or sequence of HMGB1, Dotted grey line; unknown sequence of HMGB1.

The human HMGB1 protein has 215 amino acid (aa) residues (MW: 25–30 kDa) that form two homologous DNA-binding domains (A-box, 1–79 aa; B-box, 89–162 aa) and a negatively charged C-terminal acidic tail (186–215 aa; Figure 1A) (9, 13). HMGB1 is located in the nucleus as a result of bipartite nuclear localization signals (NLS; NLS1, 28–44 aa; NLS2, 179–185 aa) mediated by the nuclear importin karyopherin (KAP)-α1; however, the affinity between the two molecules is decreased by HMGB1 phosphorylation (14, 15). Conversely, the DNA-binding domain of HMGB1 contains a nuclear-export signal (NES), and its cytoplasmic localization is mediated by the nuclear exportin chromosome-region maintenance 1 (CRM1) (16). The acidic C-terminal of HMGB1 regulates DNA binding and bending by interacting with its DNA-binding domains (8, 17) or histones H1/H3 (18); thus, HMGB1 lacking the C-terminal domain displays improved DNA looping and binding abilities (19).

The C-terminal acidic domain of HMGB1 also functions as a transcriptional activator (20, 21), while HMGB1 B-box has been reported to induce pro-inflammatory signals upon extracellular stimulation, and the A-box induces antagonistic effects (22). In particular, the 201–205 aa residues in the C-terminal acidic tail play a crucial role in the antibacterial activity of HMGB1 (23). Moreover, interactions with diverse receptors, extracellular partners, and intracellular partners play important roles in the activity and biological functions of HMGB1. HMGB1 residues 89–108 bind to Toll-like receptor (TLR) 4 and increase pro-inflammatory signaling (22), whereas residues 150–183 interact with the receptor for advanced glycation end products (RAGE) to regulate cell migration (24) and stimulate inflammation (25). HMGB1 has also been shown to bind to dendritic cell (DC)-derived TIM-3 and suppress nucleic acid-mediated innate immune responses (26). In addition, residues 1–15 and 80–96 have been found to inhibit lipopolysaccharide (LPS)-induced cytokine production in a subclinical endotoxemia mouse model (27). HMGB1 binds to lipoteichoic acid (LTA) and enhances proinflammatory responses by mediating the transfer of LTA to CD14 and TLR2 (28). Furthermore, HMGB1 residues 6–12 are responsible for binding heparin and compete with binding between RAGE and HMGB1 (29). A- and B-box of HMGB1 bind to C1q, but only B-box of HMGB1 can induce the complement activation leading to sterile inflammation (30). In addition, complex formation between HMGB1 and IL-1β enhances inflammation and destruction mechanisms in arthritic joints (31), whereas the HMGB1 and C-X-C motif chemokine ligand 12 (CXCL12) complex binds to C-X-C chemokine receptor 4 (CXCR4) and promotes the recruitment of inflammatory cells (32). Extracellular HMGB1 binds to single-stranded oligonucleotides, forming HMGB1-5'-C-phosphate-G (CpG)-DNA complex, interacting with TLR9 to augments cytokine production (33, 34). Also, HMGB1 released from apoptosis binds to the nucleosomes and induces cytokine production or dendritic cells (DCs) activation through interaction with TLR2 (35). In contrary, HMGB1-CD24 complex selectively represses the tissue damage-induced inflammation via interaction with Siglec-10 protein (36). Cytoplasmic HMGB1 binds to Beclin 1 using intramolecular disulfide bridge (Cys23 and Cys45) to affect autophagosome formation (37). Cys23 or Cys45 in HMGB1 can also bind to reactive cysteine residues in peroxiredoxins I and II (PrxI/II) to form intramolecular disulfide bonds that promote its secretion in response to inflammatory stimuli (38). Residues 7–74 are responsible for binding the p53 transactivation domain and thus increasing gene transcription (39) (Figure 1A).

Studies have modified HMGB1 A-box and B-box structures using the PyMol program based on 2YRQ [Protein Data Bank (PBD) ID: 2yrq]. These two DNA-binding domains consist of three alpha helices (helix-I, -II, and -III) and two loops (loop-I and -II) that form an L-shaped structure (40). HMGB1 binds to the minor groove of pre-bent or linear DNA with little sequence specificity (41, 42); however, both A- and B-box have the remarkable ability to unwind and bend DNA with different properties. For instance, the A-box domain recognizes pre-bent or linear DNA, whereas the B-box domain binds to mini-circles and bends linear DNA (43–45). In the crystal structure of HMGB1 showing A-box domains and an AT-rich DNA fragment, the two HMGB1 A-box domains were found to collaborate in order to interact with pre-bent or kinked DNA. The Phe37 (Phe38 in HMGB1 described here) residues from both domains were shown to play important roles in initiating intercalation with CG base pairs and thus generating highly kinked DNA (Figure 1B) (46). The B-box domain is structurally similar to the A-box in its DNA-binding characteristics and its Ile34 (Ile35 in B-box or Ile122 in the full HMGB1 sequence described here) residue is sterically comparable to the Phe37 residue in the A-box domain (Figure 1B).

HMGB1 senses and coordinates the cellular stress response. As mentioned earlier, HMGB1 contains three conserved cysteines: Cys23, Cys45, and Cys106 (Figure 1A). Cys23 and Cys45 can form an intramolecular disulfide bond depending on the reactive oxygen species (ROS) concentration and environmental conditions under which HMGB1 binds to its ligands (Figure 1C) (47). Indeed, the half-life of all-thiol-HMGB1 ranges from ~17 min in human serum and saliva to 3 h in cell culture medium (47). The oxidation state of HMGB1 determines its interactions with diverse receptors (32, 48) and its DNA-binding affinity (49). Depending on its redox status, extracellular HMGB1 can trigger numerous effects: (1) all-thiol-HMGB1 can exert chemoattractive effects by binding to CXCR4; (2) all-thiol-HMGB1 can prompt autophagy by binding to RAGE (50); (3) disulfide-HMGB1 can exert pro-inflammatory effects by binding to TLR4; and (4) fully oxidized-HMGB1 is inert. Cys106 plays a crucial role in the translocation of HMGB1 from the nucleus to the cytosol (51). Moreover, C23-C45 oxidation induces a shift between helix I and helix II in the A-box domain that reduces DNA binding affinity by altering the orientation of Phe37 (52), resulting in cytoplasmic translocation. HMGB1 can also affect transcription in the nucleus, requiring rapid transition between the all-thiol-and disulfide forms of HMGB1 (53).

Purification of HMGB1 under its native conditions yields both homodimers and oligomeric forms of the protein; however, these forms are dissociated when acid-extracted (54). Our group has also described the Cys106-mediated formation of HMGB1 dimers under conditions of excessive ROS generation at the cellular level (unpublished data). As previously discussed, HMGB1 is a versatile molecule because of intra- and inter-molecular interactions in its different domains; moreover, the protein can be either actively secreted by activated immune cells or passively released due to necrotic cell death where it acts as a damage-associated molecular pattern (DAMP). Because of its versatile and variable nature, it is important to understand the mechanism underlying the secretion of HMGB1 to fully appreciate its therapeutic and pathological potential. In this review, we briefly summarize the conventional and non-conventional mechanisms of cytokine secretion, and describe in detail the mechanisms of HMGB1 oxidation and secretion that have been determined so far, with a focus on immunological function.

## Conventional and Non-Conventional Cytokine Secretion Mechanisms

Most soluble secretory proteins utilize a well-known conventional secretion system involving the endoplasmic reticulum (ER) and Golgi network, and contain a signal peptide to target them to the ER (55). When such proteins are synthesized in the ribosome, the signal peptide is recognized by a signal recognition particle (SRP) complex. The protein-ribosome-SRP complex then moves to the Sec61 translocon complex in the ER outer membrane and proteins translocate into the ER lumen via the translocon complex (56, 57). Within the ER lumen, the protein meets chaperone proteins such as Bip and undergoes modification (glycosylation) followed by protein folding (58, 59). These proteins are then translocated to the Golgi and plasma membrane via several processes, concluding the conventional protein secretion pathway. A recent study found that when the ER/Golgi pathway is blocked, some proteins are secreted via an independent mechanism. These proteins lack a signal peptide targeting them to the ER and are secreted under specific conditions, such as ROS accumulation, inflammation, and cell growth factors. These forms of protein secretion are considered unconventional, and the secreted proteins are generally involved in immune surveillance, cell survival, and cellular stress (60).

Cytokines regulate immunological functions via their secretion in immune environments; therefore, controlling cytokine secretion is crucial for regulating immune function. Many cytokines such as tumor necrosis factor-α (TNF-α), interleukin (IL)-2, and IL-12 are secreted via the conventional secretion mechanism; however, some, including HMGB1, IL-1β, fibroblast growth factor (FGF)-1/2, and galectins, utilize non-conventional secretion mechanisms. Here, we briefly explore different cytokines that use various secretion mechanisms, and summarize them in Table 1.

**Table 1:** Conventional and non-conventional secretion of cytokines.

**Cytokine**	**Conventional or Non-conventional**	**Secretion mechanism**	**References**
TNF-α	Conventional	- Translocated across the ER and through the Golgi apparatus to the plasma membrane	(61, 62)
IL-1β	Non-conventional	- Secretion by secretory lysosome, microvesicles shed, or exosome - Gasdermin D (GSDMD)-dependent	(63–67)
FGF-1/2	Conventional/Non-conventional	- FGF-1/2 are not only secreted via the conventional secretion pathway, but also the non-conventional secretion system FGF-1 - Secretion is increased by cellular stresses such as heat shock, hypoxia, and serum starvation FGF-2 - Dependent on forming complexes with Na^+^/K^+^-ATPase	(68–73)
Galectins	Non-conventional	- Accumulate at the plasma membrane and induce the formation of exosomes pinched off and released into extracellular space	(74–76)

### Tumor Necrosis Factor (TNF)-α

TNF-α was first identified for its anti-tumor activity but is now also known to act as a multifunctional cytokine in host defense mechanisms during inflammatory responses (77). Since it contains an ER signal peptide, TNF-α is translocated across the ER and through the Golgi apparatus to the plasma membrane (61). Newly synthesized TNF-α is localized in the trans-Golgi network (TGN) with golgin and p230/golgin-245 for intracellular trafficking and cell surface delivery (78). TNF-α is then released from the granule via fusion with the plasma membrane (62) and is cleaved at the cell surface by the tumor necrosis factor-α converting enzyme (TACE) between Ala^76^ and Val^77^ (79).

### Interleukin (IL)-1β

IL-1β plays important roles in the cytokine response to inflammation and immunity during bacterial or viral infection and is mainly secreted by monocytes, macrophages, and CD in response to inflammasome activation under conditions such as LPS and adenosine triphosphate (ATP) stimulation (80). Although IL-1β is passively released during pyroptotic cell death (63), secretion of cleaved-IL-1β requires cytosolic compartments such as the secretory lysosome (64), microvesicles shed from the plasma membrane (81) or exosomal release (65). Previous studies have reported that caspase-1/11 cleave gasdermin D (GSDMD), whose N-terminal then forms pores in the plasma membrane that are crucial for the passive release of IL-1β during pyroptosis (66, 67). Although the pathway is unknown, IL-1β secretion is known to require ABC transporters whose knockdown or inhibition is reported to ameliorate IL-1β secretion (82, 83).

### Fibroblast Growth Factor (FGF) -1 and -2

FGF-1 and -2 belong to a family of heparin-binding growth factors (84) and control mitogenic activity (85) and tumor-induced angiogenesis (86). FGF-1/2 are not only secreted via the ER/Golgi-dependent secretion pathway, but also the non-conventional secretion system (68–70); however, the secretion pathways of FGF-1 and FGF-2 are distinct (87). Unlike FGF-1, FGF-2 secretion is sensitive to Na^+^/K^+^-ATPase inhibition and is dependent on forming higher-order complexes with Na^+^/K^+^-ATPase ion transporters, with its export occurring in a membrane potential-independent manner (71). Conversely, FGF-1 secretion is increased by cellular stresses such as heat shock (88), hypoxia (72), and serum starvation (73), while copper also can induce FGF-1 secretion by forming multiprotein aggregates in response to stress (89); however, FGF1 folding does not prevent its export (90).

### Galectins

Galectins are a family of abundant β-galactoside-specific lectins that reside in the extracellular matrix and are implicated in many cellular processes, such as proliferation, differentiation, and apoptosis (91, 92). Since galectins lack the signal peptides found in IL-1β and FGF-1/2 for ER/Golgi-mediated secretion, their secretion is not blocked by the ER/Golgi-dependent inhibitors brefeldin A and monensin (74, 93). Moreover, galectin-1/3 are not packaged into vesicles during extracellular export (74, 75, 94, 95) but accumulate at the plasma membrane and induce the formation of exosomes that are pinched off and released into extracellular space (74, 75, 94, 95). Secreted galectins bind to the extracellular surface of the plasma membrane or extracellular matrix (75, 76) via the *N-* and *O-*glycosylated β-galactose-terminated oligosaccharide side chains of glycoproteins (74, 92).

## HMGB1 Secretion

**Figure 2:**
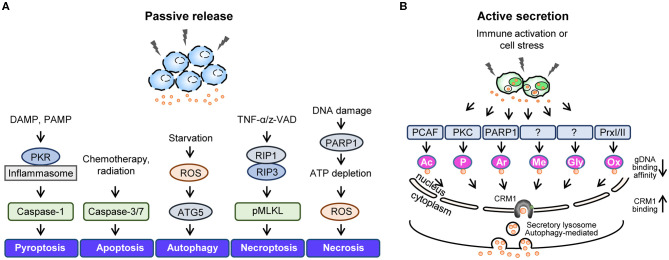
Active secretion and passive release of HMGB1. Summary of stimuli leading to passive HMGB1 release **(A)** and active HMGB1 secretion **(B)**. **(A)** Passive release mechanisms involve the disruption of the plasma membrane via various cell death mechanisms. **(B)** Active secretion involves various HMGB1 post-translational modifications that reduce its genomic DNA binding activity and increase its CRM1 binding affinity. DAMP, danger-associated molecular pattern; PAMP, pathogen-associated molecular pattern; PKR, double-stranded RNA-dependent protein kinase; ROS, reactive oxygen species; ATG5, autophagy related 5; z-VAD, carbobenzoxy-valyl-alanyl-aspartyl-[O-methyl]; RIP1, receptor-interacting serine/threonine-protein kinase 1; RIP3, receptor-interacting protein kinase 3; pMLKL, Phosphorylated mixed linage kinase domain like; PARP1, poly [ADP-ribose] polymerase 1; PCAF, P300/CBP-associated factor.

Generally, cytokines containing a leader sequence undergo secretion via the ER/Golgi secretion pathway; however, the non-histone nuclear protein HMGB1 lacks this signal peptide, and studies have suggested that HMGB1 secretion involves diverse unconventional secretion pathways. For instance, infection or cellular stress has been shown to increase the cytoplasmic accumulation of HMGB1, which is then passively released into the extracellular space or actively secreted via secretory lysosomes (9) (Figure 2). Here, we describe in detail the mechanisms that participate in HMGB1 secretion.

### Passive Release of HMGB1

HMGB1 can be passively released during various forms of cell death, including pyroptosis, apoptosis, autophagy, necroptosis, and necrosis (Figure 2A). Pyroptosis refers to inflammatory programmed cell death that occurs after inflammasome formation caused by bacterial or viral infection. During pyroptosis, double-stranded RNA-dependent protein kinase (PKR) induces inflammasome formation, caspase-1 activation, and HMGB1 release upon exposure to diverse inflammasome-activating agents (96). Apoptosis is another form of programmed cell death that occurs when cells die due to injury and involves caspase-3/7, which belong to a family of protease enzymes. Apoptotic cells induce HMGB1 release, and it has been reported that Z-VAD, a pan-caspase inhibitor, can reduce the levels of HMGB1 released (97). Autophagy is an intracellular degradation system that balances energy sources in response to nutrient stress by regulating the degradation of cellular material using lysosomes or vacuoles; however, excessive autophagy can lead to cell death. Indeed, studies have shown that epithelial and glioblastoma tumor cells release HMGB1 when treated with the autophagy-inducing agent epidermal growth factor receptor-targeted diphtheria toxin (DT-EGF) (98), also ATG5 knock-out bone marrow-derived macrophages (BMDMs) reduced HMGB1 secretion under EBSS starvation conditions (99). In addition, autophagosome-mediated HMGB1 secretion has been identified, with ATG5 deficient cells or those treated with an early autophagy inhibitor displaying all-thiol-HMGB1 secretion (data not published). Necrosis is a form of premature cell death caused by the loss of membrane integrity, intracellular organelle swelling, and ATP depletion, and it has been shown that HMGB1 is passively released by necrotic or damaged cells (100). Necroptosis is a form of programmed necrosis mediated by death signals that cause the phosphorylated mixed lineage kinase domain-like protein (MLKL) to be inserted into and permeabilize the plasma membrane (101). Moreover, TNF-α/Z-VAD-induced necroptosis has been shown to phosphorylate MLKL proteins and increase HMGB1 secretion levels (102).

### Post-Translational Modifications (PTMs) and Active Secretion of HMGB1

HMGB1 can undergo several extensive PTMs that increase its cytoplasmic accumulation and extracellular secretion during infection or cell stress, including acetylation (16), phosphorylation (14, 15), ADP-ribosylation (103), methylation (104), glycosylation (105), and oxidation (38, 51) (Figure 2B). Various PTMs increase the interaction between HMGB1 and the nuclear transport receptor CRM1, thus favoring its translocation from the nucleus to the cytoplasm. PTM-mediated HMGB1 secretion is caused by lysosomal exocytosis wherein cytoplasmic HMGB1 co-localizes with the lysosomal marker LAMP1 for secretion (106). The PTMs and subsequent events that HMGB1 undergoes are summarized below and visualized in Figure 2B.

1. Acetylation is a major PTM that can affect protein function by altering properties such as hydrophobicity, solubility, and surface properties. Protein acetylation refers to the reaction during which the acetyl group of acetyl coenzyme A (Ac-CoA) is transferred to the lysine (Lys) residue of the target protein. HMGB1 has two acetylation clusters at Lys27–29 and Lys181–183, and it has been shown that nuclear localization is unaffected by mutating either Lys cluster (16). The poly(ADP-ribose) polymerase-1 (PARP1) induces the cytoplasmic translocation and extracellular secretion of HMGB1 by catalyzing its acetylation (107). Compare to HMGB1, mimicking acetylated HMGB1 (six lysine residues for glutamines) increases the TNF-α production in RAW264.7 cells and reduces DC maturation (108). Various triggers which induces HMGB1 acetylation includes inflammatory signal such as LPS or TNF-α (109), and cell stress triggered by chemotherapeutic reagent such as cisplatin (110). Such conditions can be experimentally mimicked using trichostatin A (TSA), an inhibitor of histone deacetylase complex (HDAC) (111).
2. Phosphorylation is a molecular mechanism via which amino acid residues are phosphorylated by a protein kinase to regulate the functional response of proteins to various extra- or intracellular stimuli. HMGB1 phosphorylation is mediated by classical protein kinase C (cPKC) in a calcium-dependent manner via the PI3K-PKC signaling pathway (14). In HMGB1, Ser35, 39, 42, and 46 in NLS1, 181 in NLS2, and 53 close to NLS1 have been shown to be phosphorylated in macrophages after TNF-α and okadaic acid treatment (15), while Ser39, 53, and 182 of HMGB1 are phosphorylated by PKC-ζ in colon cancer cells (112). Moreover, HMGB1 phosphorylation has been found to reduce its binding affinity with the nuclear import protein KAP-α1 and promote its cytoplasmic translocation and extracellular secretion (15).
3. ADP-ribosylation refers to the process wherein one or more ADP-ribose moieties are added to target proteins, and includes mono-ADP-ribosylation, poly-ADP-ribosylation, ADP-ribose cyclization, and *O*-acetyl-ADP-ribose formation. PARP activation regulates the translocation of HMGB1 from the nucleus to the cytoplasm during DNA-alkylating damage (103), while hyper poly(ADP)-ribosylated HMGB1 has been shown to inhibit efferocytosis by binding to phosphatidylserine (PS) on apoptotic cells and RAGE on macrophages (113). Such activation was reported in cell death related stimuli such as activation of tumor necrosis factor [ligand] superfamily member 10 (TNFSF10)—TNF-related apoptosis-inducing ligand (TRAIL) pathway (114) or daunorubicin treatment (115), and inflammatory assault with LPS (103).
4. Methylation is a PTM in which a methyl group is added to proteins, usually on the side-chain nitrogens of arginine and lysine or carboxyl groups of glutamate and leucine. During the process of neutrophilic differentiation, Lys42 in HMGB1 can be mono-methylated which significantly reduces its DNA binding activity, causing its translocation from the nucleus to the cytoplasm (104). Lys112 has also been found to be mono-methylated in HMGB1 and contribute toward its cytoplasmic localization (116). However, it is still unclear which stimuli exclusively leads to the methylation of HMGB1 during active secretion.
5. Glycosylation is a common PTM characterized by the attachment of sugar moieties to proteins. HMGB1 derived from calf thymus and chicken erythrocytes undergoes *O*-linked GlcNac glycosylation with sugars such as Fuc, Man, GalNH_2_, GlcNH_2_, and Gal monosaccharides (117), while it has recently been reported that HMGB1 can also undergo *N*-linked glycosylation at two consensus (Asn37 and Asn137) residues and one non-consensus (Asn135) residue. *N*-glycosylation of HMGB1, induced by PMA, TSA and LPS, can persuade its secretion into the extracellular space by reducing its DNA binding affinity and increasing the association with CRM1 (105).
6. Oxidation is a covalent modification that proteins undergo during redox reactions involving the transfer of oxygen, hydrogen, and electrons. HMGB1 contains three redox-sensitive cysteines: Cys23, Cys45, and Cys106. Under mild oxidative stress, Cys23 and Cys45 rapidly form an intramolecular disulfide bond that increases the cytoplasmic localization and extracellular secretion of HMGB1 (51). In response to inflammatory stimuli, PrxI and PrxII induce HMGB1 oxidation to its disulfide form and lead to its nucleocytoplasmic translocation and secretion (38). Such oxidative stresses may come from external sources such as H_2_O_2_ or glucose oxidase, or by internal activation of molecules by LPS, TNF-α, or interferon-γ which in turn causes HMGB1 release through TNF-dependent manner (118). Since the oxidation status and immunological properties of HMGB1 crucially influence its biological function, we dedicated a separate section to discussing HMGB1 redox biology in detail.

### HMGB1 Oxidation Mechanisms

**Figure 3:**
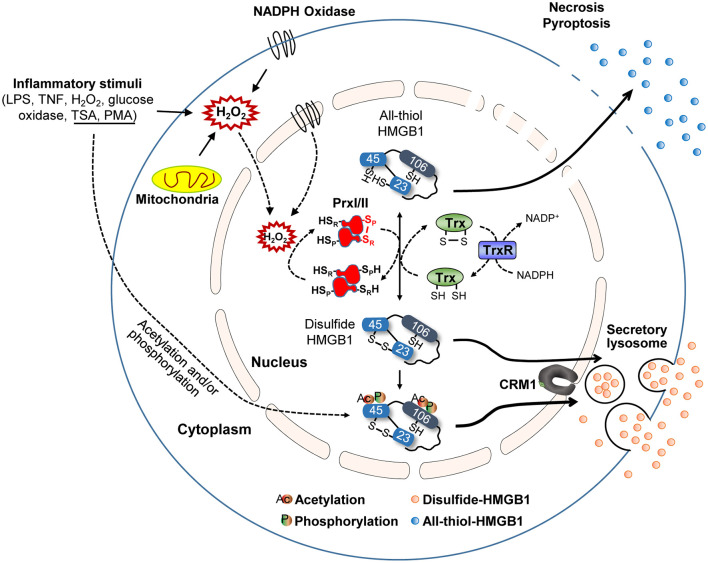
HMGB1 redox biology. Summary of HMGB1 redox biology and the crucial role of peroxiredoxin and thioredoxin. Various stimuli cause oxidative stress that promotes HMGB1 oxidation via the peroxiredoxin-dependent pathway. NADPH, nicotinamide adenine dinucleotide phosphate; S_P_, peroxidatic cysteine; S_R_, resolving cysteine; H, hydrogen; Trx, thioredoxin; TrxR, thioredoxin reductases; NADP, nicotinamide adenine dinucleodebtide phosphate.

The function and secretion of HMGB1 are dependent on its redox status, which is controlled by three redox-sensitive cysteines: Cys23 and Cys45 in A-box and Cys106 in B-box. Thus, HMGB1 can take three different oxidation forms: an all-thiol form, disulfide form (Cys23-Cys45 intramolecular disulfide bond with Cys106 thiol form), and a fully oxidized form. During the active secretion of HMGB1, the Cys23-Cys45 intramolecular disulfide bond induces HMGB1 cytoplasmic translocation. This process also requires Cys106, as demonstrated by the nuclear localization of Cys106-mutated even with Cys23 and Cys45 mutations (51). Hydrogen peroxide is a ROS that induces the release of HMGB1 from macrophages and monocytes, reportedly by increasing its interaction with CRM1 and thus increasing HMGB1 secretion (119). Under elevated ROS conditions, PrxI and PrxII cause disulfide-HMGB1 formation (38) (Figure 3). The diversity of HMGB1 redox status also affects its passive release from necrotic and apoptotic cells, with the majority of HMGB1 released from necrotic cells being in an all-thiol state but that released from apoptotic cells being in a fully oxidized form. Moreover, HMGB1 oxidation status plays an important role in receptor binding and subsequent cytokine-like activities.

Hoppe et al. previously described the mechanism of HMGB1 oxidation (51), identifying that HMGB1 interacts with the de-glutathionylation enzyme glutaredoxin (Grx) during the nuclear extraction of Chinese hamster ovary (CHO) cells after diamide treatment. Electrophoretic mobility assays revealed that HMGB1 oxidation increases in a diamide concentration-dependent manner, while disulfide-HMGB1 could be reversed by incubating diamide-treated retinal pigment epithelium (RPE) cells with thioredoxin (Trx) or Grx/glutathione (Figure 3). Conversely, we found that HMGB1 can be oxidized by PrxI/II in the nucleus after exposure to inflammatory stimuli (Figure 3) (38). PrxI/II can interact with all-thiol-HMGB1 generated by mutagenesis (Cys^23^-to-Ser or Cys^45^-to-Ser) after hydrogen peroxide stimulation; however, such HMGB1 oxidation is suppressed in PrxI/II-deficient mouse embryonic fibroblast (MEF) cells, even when exposed to inflammatory stimuli. All-thiol-HMGB1 cannot translocate into the cytoplasm or extracellular space, while PrxI/II-deficient BMDMs lack the ability to secrete HMGB1 despite treatment with diverse inflammatory stimuli, such as LPS, phorbol-12-myristate-13-acetate (PMA), trichostatin A (TSA), or TNF-α (38). HMGB1 phosphorylation by PMA and/or acetylation by TSA promotes its nuclear transport and extracellular secretion (15, 16). Although cells treated with PMA or TSA display increased HMGB1 secretion, this secretion is inhibited by treatment with the antioxidant *N*-acetylcysteine (NAC). Moreover, HMGB1 secretion induced by PMA can be inhibited by the NADPH oxidase inhibitor diphenyleneiodonium (DPI), indicating that PMA-induced HMGB1 secretion requires H_2_O_2_ production by the mitochondria and/or Nox. Intracellular disulfide bond formation in HMGB1 (Cys^23^-Cys^45^) has been shown to be important for its nucleocytoplasmic translocation and extracellular secretion. Indeed, HMGB1 mutants with defective phosphorylation or acetylation sites undergo less translocation into the extracellular space than WT HMGB1 (38). In summary, these findings indicate that the mechanisms of HMGB1 oxidation and reduction are induced by PrxI/II and Trx or Grx/glutathione, respectively (Figure 3). It is possible that, like acetylated-HMGB1, oxidized-HMGB1 may be less favored for nuclear import and thus accumulates in the cytosol. Oxidized-HMGB1 in the cytosol is packed into lysosomes through an as yet unknown mechanism and then secreted. Nevertheless, extracellular HMGB1 can induce an immune response and HMGB1 oxidation decides its immune function. Also, as mentioned previously, HMGB1 oxidation has a more substantial influence on its secretion compared to acetylation and phosphorylation. Thus, control of HMGB1 oxidation both in intracellular and extracellular is important for the therapeutic approach based on blockade of HMGB1 secretion and immune response.

### HMGB1 Secretion Kinetics

**Figure 4:**
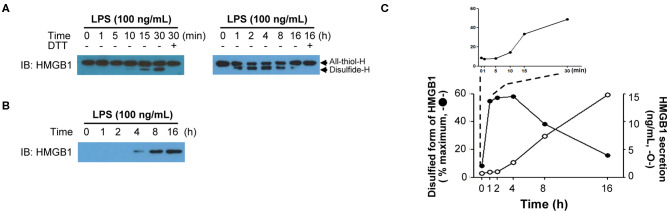
HMGB1 redox kinetics. **(A)** Short-term (left) and long-term (right) changes of HMGB1 redox status upon LPS stimulation. Western blot showing the All-thiol-H (all-thiol-HMGB1) or Disulfide-H (disulfide-HMGB1) expression in BMDM whole-cell lysates, which was treated with LPS (100 ng/mL) for indicated times. Methods were used as our previous study (38). **(B)** HMGB1 secretion timeframe. Disulfide-H from culture supernatant was measured by Western blot. **(C)** Graphical representation of the relationship between HMGB1 oxidation (left *y*-axis, closed circle) quantified as % maximum from **(A)** and HMGB1 secretion (right *y*-axis, opened circle). The level of secreted HMGB1 was determined by ELISA (38).

The PTMs of HMGB1 in the nucleus occurs rapidly after exposure to diverse stimuli; however, after binding to CRM1 in the nucleus, the export of modified HMGB1 into the cytoplasm is known to take around 6–8 h, whereas it can take up to 18 h for HMGB1 secretion into the extracellular space to peak. The degree of HMGB1 oxidation has been reported to be crucial for its immune function (9, 51, 120) and is also very important for its secretion. For instance, HMGB1 secretion induced by PTMs such as phosphorylation or acetylation also requires oxidation, with anti-oxidant treatment reducing HMGB1 secretion even when treated with PMA or TSA (38). Therefore, we examined instances when HMGB1 PTMs and extracellular secretion occur under oxidative conditions by treating mouse BMDMs with 100 ng/mL of LPS and separating their nuclei at a series of time points to determine the HMGB1 oxidation ratio. HMGB1 oxidation increased with time, with oxidation first detectable after just 30 min, and disulfide-HMGB1 was maintained for up to 4 h and then gradually decreased after 8 h. Despite rapid HMGB1 oxidation in the nucleus, its secretion began only after 4 h and increased up to 16 h (Figure 4). Further studies investigating the mechanism underlying the delay between oxidation and secretion would improve our understanding of HMGB1 secretion kinetics. It is possible that secretion-ready cytosolic HMGB1 is packed into a secretory lysosome or autophagosome and secreted via non-conventional secretion mechanisms, requiring a very complex, as yet unknown, packaging mechanism. Conversely, a recent article by Wang *et al*. observed HMGB1 localization in mitochondria and peroxisome in neuron cells via electron microscopy and immunofluorescence, but not in lysosomes (121). In macrophages or macrophage lineages, the release of HMGB1 occurred through a lysosomal pathway after acetylation of the HMGB1 (16). It has also been demonstrated that LPS-induced HMGB1 secretion by monocytes is mediated by lysosomal exocytosis (106). Various explanations may be available for such discrepancy; however, the diverse origin of cells (macrophage, epithelial, neuronal, etc.) may have different mechanisms that translocated HMGB1 takes to be secreted to the extracellular milieu. A complete understanding of the oxidation kinetics mechanism of HMGB1 is necessary to predict the fate of HMGB1-mediated inflammation.

### HMGB1 Receptors and Immune Functions

**Figure 5:**
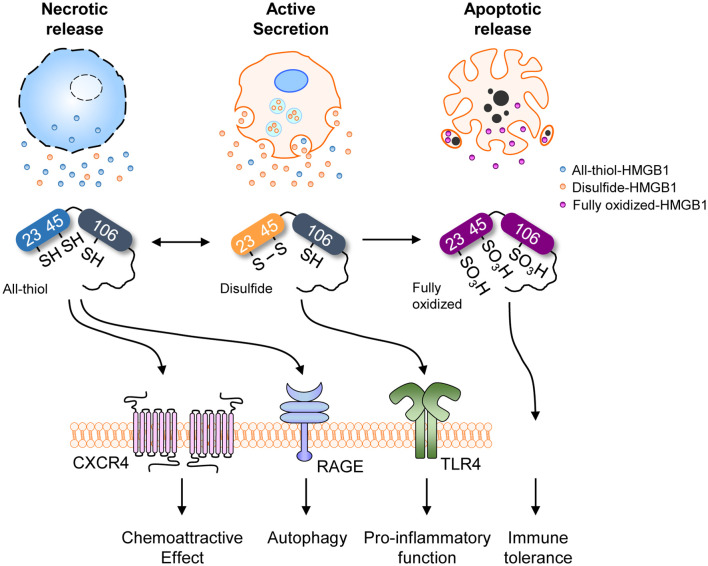
HMGB1 redox status and receptors. Redox status of HMGB1 from various sources with different receptors and their representative functions. Different redox states are associated with different release mechanisms, each linked to various immunological and cell biological functions.

As mentioned in the Introduction, extracellular HMGB1 via active secretion or passive release binds to diverse partners (Figure 1A). HMGB1 associates and shows interactions with several molecules, such as Heparin, LPS, LTA, IL-1β, RNA, and DNA, CXCL12, nucleosomes and C1q (9, 27–30). HMGB1-partner complex (or HMGB1 alone) interacts with immune receptors or surface molecules such as TLRs and RAGE, which then activate the downstream signaling pathways such as MyD88, IFN regulatory factors (IRFs), nuclear factor κB (NF-κB), MAPKs and phosphatidylinositol 3-kinase (PI3K) to enhance the inflammation and immune response (9, 22, 25, 26, 28, 34, 36). Besides, HMGB1 oxidation status alters its receptor bindings and subsequent cytokine-like activities (9, 51). As mentioned in section Post-Translational Modifications (PTMs) and Active Secretion of HMGB1, multiple PTMs are involved in extracellular secretion of HMGB1. Effect of PTM in receptor-ligand interaction kinetics between extracellular HMGB1 and its receptors, however, are understood to a lesser degree. One example of PTMs positively affecting the HMGB1-receptor interaction is portrayed using acetylation-mimicking mutant HMGB1 (six lysine residues for glutamines), increasing TNF-α production in RAW264.7 cells (108). Thus, although specific effects for each PTM are yet to be reported, diversely modified HMGB1 may have an important role in the receptor binding and downstream signaling pathway. Of all PTMs associated with HMGB1, only the effects of oxidation are studied in detail, hence we will focus our scope to the redox status of extracellular HMGB1 in regards to its activity. HMGB1 binds to different receptors depending on the redox state of its three cysteines (C23, C45, C106), which subsequently determines its functions (120). HMGB1 containing three thiol-form cysteines exerts chemoattractive effects by forming a heterocomplex with CXCL12, which then binds to CXCR4 and induces cell migration. The formation of a complex between HMGB1 and CXCR4 induces conformational changes in aa residues 3–12 of CXCL12 alongside specific conformational changes in the CXCR4 homodimer, which promotes better chemotactic abilities than CXCL12 alone (32). All-thiol-HMGB1 can also bind to RAGE and promote autophagy (122) by inhibiting mTOR and promoting Beclin 1-Ptdlns3KC3 complex formation (123). ROS induces HMGB1 oxidation and cytosolic translocation from the nucleus. Cytoplasmic HMGB1 binds to Beclin 1 using an intramolecular disulfide bridge (Cys23 and Cys45) to enhance autophagic flux (37, 124, 125). Moreover, the interaction between HMGB1 and RAGE activates NF-κB, the MAP kinase pathways and affects cell migration by inducing the expression of adhesion molecules (126, 127). Extracellular HMGB1 can also stimulate RAGE expression (128). Conversely, disulfide-HMGB1 stimulates cytokine production and inflammation by forming a complex with CD14 and MD-2 via TLR4. The disulfide bond between C23-C45 and the thiol form of C106 residues are not only required for binding TLR4 but also inducing the translocation of NF-κB and release of TNF-α (48). These findings were confirmed in apoptotic cells where fully oxidized-HMGB1 produced by excessive ROS contributed toward immunological inertness and apoptotic cell death (32). Although its specific functions remain unclear, fully oxidized-HMGB1 is known to prevent the cytokine or chemokine activities of other HMGB1 forms and ultimately induce immune tolerance (32) (Figure 5). In contrast, CD24-Siglec-10 and TIM-3 are negative receptors that inhibit HMGB1 immune activity in macrophages, DCs and tumor cells. HMGB1 can bind to CD24, first identified as a B cell differentiation marker, and selectively represses the tissue damage-induce inflammation through induction of CD24-Siglec-10 complex formation, negatively regulating NF-κB (36). Also, HMGB1 can bind to TIM-3, a member of the T-cell immunoglobulin domain and mucin domain family, and its binding suppresses the nucleic acid-mediated antitumor immunity via A-box competing with nucleic acid (26). The aforementioned reports indicate extracellular HMGB1 have not only pro-inflammatory effects but also anti-inflammatory effects according to the microenvironment.

Extracellular HMGB1 not only differ in functions by its PTM derivations, but also by the types of cells responding to HMGB1. Monocytes exposed to HMGB1 polarized toward pro-inflammatory (M1) macrophages, upregulating the production of inflammatory cytokines both *in vitro* and *in vivo* (129, 130). Silencing of HMGB1, on the other hand, prevents macrophage polarization to the M1 phenotype following LPS stimulation (131). Similarly, neutrophils react to extracellular HMGB1 by promoting its neutrophil extracellular trap formation (132) and heighten its immune reactions (133). DCs consider HMGB1 as an endogenous adjuvant to boost its effectiveness in antigen-presenting to its adaptive counterparts (134). Extracellular HMGB1, as discussed above, has exhibited significance in various immunological and physiological contexts, sparking an interest in suppressing its functions. Controlling HMGB1 as a potential therapeutic target in the immune diseases must be exquisitely controlled depending on its purpose.

### HMGB1 Inhibition

**Figure 6:**
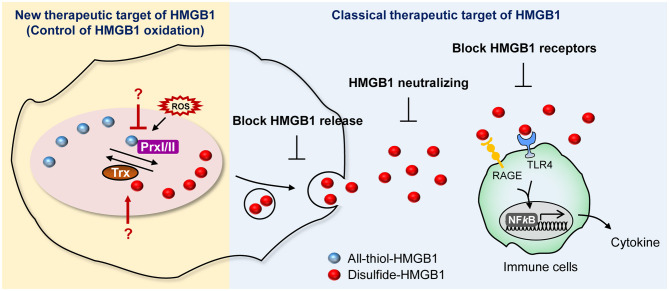
Current and potential strategies for HMGB1 inhibition. Summary of newly proposed inhibition targets (left), targets utilized by conventional inhibitors (right).

The involvement of HMGB1 in various pathologies ranging from inflammatory diseases to cancer has been discussed thoroughly and has resulted in the development of HMGB1 secretion inhibitors. Currently, several companies and research centers sought to control the effects of HMGB1 by modulating its expression, translocation, secretion, and receptor binding ability using diverse chemicals as an approach to develop therapeutic agents. These strategies for suppressing HMGB1 secretion can be divided into three categories: (1) small molecules inhibiting HMGB1 release; (2) neutralizing HMGB1 itself; and (3) blocking HMGB1 receptors (Figure 6).

Numerous studies have reported small molecules capable of inhibiting HMGB1 secretion, from newly synthesized molecules to those isolated from natural sources. For instance, naturally isolated small molecules such as glycyrrhizin have been reported to be effective in treating numerous pathological conditions, such as septic shock, neuroinflammation, atopic dermatitis, and *Pseudomonas aeruginosa* keratitis (135–138). Synthetic molecules such as ethyl pyruvate, atorvastatin, and simvastatin have also demonstrated promising therapeutic activity by targeting HMGB1. In addition, the natural flavonoid kaempferol was found to alleviate neuroinflammation by suppressing HMGB1 release and down regulating the TLR4/MyD88 pathway (139), and the rare ginsenosides Rk1 and Rg5 have shown promise by reducing HMGB1 release and thereby improving survival in cecum ligation- and puncture-induced murine sepsis models (140). Inflachromene, a novel small molecule developed as a potential anti-inflammatory drug, was also found to inhibit HMGB1 secretion via directly binding to HMGB1 and inhibiting autophagy (141, 142). Despite most of the candidates are yet to be approved by the Food and Drug Administration (FDA), Metformin, clinically approved drug for metabolic disease and type 2 diabetes, has investigated as an inhibitor for HMGB1 through direct binding each other, inhibiting the cytosolic translocation within the cells and receptor binding in the extracellular space (143, 144). Other candidates of HMGB1 secretion inhibitors are also being discovered through drug repositioning efforts, such as salicylic acid, methotrexate, and (-)-epigallocatechin-3-gallate (145–147).

Neutralizing antibodies against HMGB1 have been used to confirm its involvement in mouse models of various pathologies, such as arthritis, suggesting that HMGB1-neutralizing antibodies could be used therapeutically (148, 149). Indeed, neutralizing the effects of HMGB1 by competitively inhibiting its activity with soluble receptors or neutralizing antibodies could be a straightforward and approach. A soluble form of RAGE was reported to effectively reduce neutrophilic asthma attacks and angiotensin II-induced cardiomyocyte hypertrophy by inhibiting the HMGB1-RAGE axis (150, 151). Similarly, studies have reported the inhibition of the HMGB1-receptor signaling pathway using neutralizing antibodies against its receptors. For instance, neutralizing monoclonal antibodies recognizing TLR4 were used to reduce IL-8 secretion upon LPS stimulation in human primary monocytes, and efforts to use HMGB1 neutralizing antibodies in stroke patients are being continually made (152, 153). Moreover, the continual administration of neutralizing antibodies against RAGE in murine models of neurological pain were reported to reverse mechanical hyperalgesia (154, 155) Further information about HMGB1 inhibitors could be found in this Frontiers Research Topics of “The Role of HMGB1 in Immunity” by Yang et al. (156).

We suggest expanding on the importance of modulating the HMGB1 oxidation mechanism. HMGB1 oxidation is the major PTM that drives secretion and its oxidized form of extracellular HMGB1 induce inflammatory signaling, which leads to many diseases, including neuroinflammation, hyperalgesia, drug-induced liver injury, and sepsis (157–159). Of particular note is the fact that it is important to develop specific inhibitors targeting the enzymes involved in altering HMGB1 redox status. Prx, which is a major direct modulator of HMGB1 redox status, could possibly be a plausible candidate for inhibition (38), whereas well-established redox enzymes with a strong connection to Prx, such as Trx and sulforedoxins, could also be potential targets for inhibition (160) (Figure 6).

## Concluding Remarks

Although several strategies have been shown successfully in inhibiting HMGB1-dependent inflammatory processes (156), there is still a lack of specificity originating from HMGB1's involvement in pathologies. This review aims to overcome the aforementioned weak points by suggesting various plausible aspects of inhibition, increasing the specificity of inhibition therapies. We provide an overview of the protein secretion mechanisms and discuss the HMGB1 secretion mechanisms and pathways in depth. Besides, we highlight the importance of multiple PTMs and the redox biology of HMGB1, with a particular focus on the important role of HMGB1 oxidation in its secretion. Finally, we discuss multiple immunological and non-immunological diseases involving HMGB1, as well as attempts to inhibit its secretion, extracellular activity, or the receptors that bind to HMGB1. The next step should to unveil fine-tuned process of HMGB1 PTMs in physiological and pathological conditions. Future researches would benefit from extensive quantitative analysis of extracellular HMGB1 and its PTM patterns in various cell types and different pathological conditions to further develop disease-specific inhibition strategies.

## Author Contributions

MK and HK wrote the manuscript, contributed to the conception, design, and analysis of the study. BL and YK contributed to interpretation of the study and drafted the manuscript. MK assisted in manuscript editing and figure preparation. MS and J-SS contributed to the conception and design of the study and the discussion, writing, supervision, and critical revision of the manuscript.

## Conflict of Interest

The authors declare that the research was conducted in the absence of any commercial or financial relationships that could be construed as a potential conflict of interest.
